# Endoscopic resection of giant gastrointestinal stromal tumor at the esophagogastric junction: a case report

**DOI:** 10.1186/s12876-019-1151-5

**Published:** 2019-12-27

**Authors:** Feng Xue, Wei Wang, Ning Shi, Xing-Bin Ma, Cheng-Xia Liu

**Affiliations:** Department of Gastroenterology, Binzhou Medical University Hospital, No. 661 Huanghe 2nd Road, Binzhou City, Shandong Province People’s Republic of China

**Keywords:** Gastrointestinal stromal tumor, Endoscopic submucosal excavation, Esophagogastric junction, Case report

## Abstract

**Background:**

Gastrointestinal stromal tumors (GISTs) at the esophagogastric junction are rare and its treatment is complicated and challenging. Endoscopic resection has advantages with less complications compared to open and laparoscopic surgery.

**Case presentation:**

We report a 33-year-old male patient who was admitted to our department complaining of abdominal fullness for 20 days. A huge submucosal tumor at the esophagogastric junction was found by upper gastrointestinal endoscopy. We successfully resected the lesion through endoscopic submucosal excavation without complications, which was pathologically confirmed to be a GIST. The patient was discharged 5 days after operation and has been doing well, and there was no recurrence 8 months after the operation.

**Conclusion:**

ESE is possibly an effective and minimally invasive method of giant esophagogastric junction stromal tumor.

## Core tip

We report a case of a giant gastrointestinal stromal tumor (GIST) at the esophagogastric junction, which was successfully resected through endoscopic submucosal excavation and confirmed pathologically. The patient has a good recovery without recurrence during the 8-month follow-up after operation.

## Background

Gastrointestinal stromal tumors (GISTs) are the most common mesenchymal tumors in the gastrointestinal tract with an estimated prevalence of 15–20 per 1,000,000 [1]. As GISTs tend to be malignant, complete resection of GISTs is recommended [2]. Open surgery and laparoscopic surgery are frequently used approaches in GISTs. However, although GISTs can be completely resected through open surgery or laparoscopic wedge resection (LWR), severe gastric structural changes and stenosis often occur after these procedures leading to gastric dysfunction, especially in GISTs in the esophagogastric junction (EGJ). The indications for endoscopic resection have been expanding in recent years. However, endoscopic resection of submucosal tumors in the EGJ is rarely reported. We here report a case of a GIST at the EGJ successfully treated by endoscopic resection without postoperative complications.

## Case presentation

**Fig. 1:**
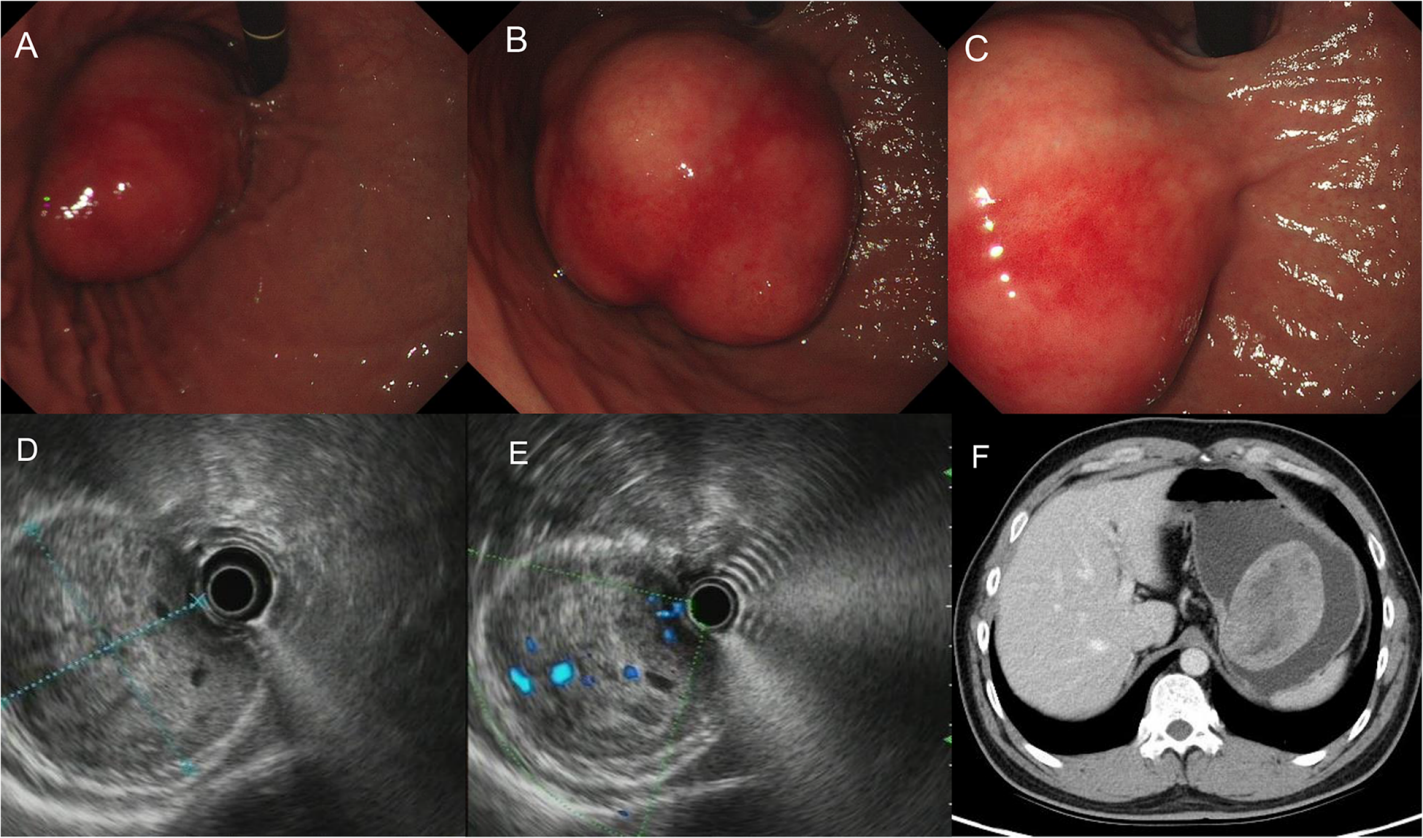
**a**, **b** and **c** Upper gastrointestinal endoscopy revealing a submucosal tumor at the lesser curvature of the esophagogastric junction. **d** and **e** Endoscopic ultrasonography revealed a round, low-echoic mass. **f** The enhanced computed tomography (CT) scan shows a mass abnormal enhancement, and the lesion is seen on the side of lesser curvature of gastric body (9.5 cm × 6.3 cm) at the esophagogastric junction

A 33-year-old man was admitted to our hospital complaining of abdominal fullness for 20 days. He had no medical history or medication. Physical examination and laboratory findings were unremarkable. Upper gastrointestinal endoscopy revealed a large submucosal eminence at the side of the lesser curvature of the stomach near the cardia, growing intraluminally without ulceration, and obstructing the gastric cavity (Fig. 1a, b, c). Endoscopic ultrasonography showed that the lesion originated from the fourth layer of the gastric wall, i.e., the muscularis propria, exhibiting nonuniform and low echo, and was about 5x7cm in diameter (Fig. 1d, e). A space-occupying lesion of about 9.5 × 6.3 cm in the small curvature of the stomach near the cardia with a nonuniformly enhanced mass was found by enhanced computed tomography (CT, Fig. 1f). CT scanning of the small intestine showed no stromal tumor. Because the stromal tumor was close to the cardia, to better protect the cardia, we performed endoscopic submucosal excavation (ESE).

**Fig. 2:**
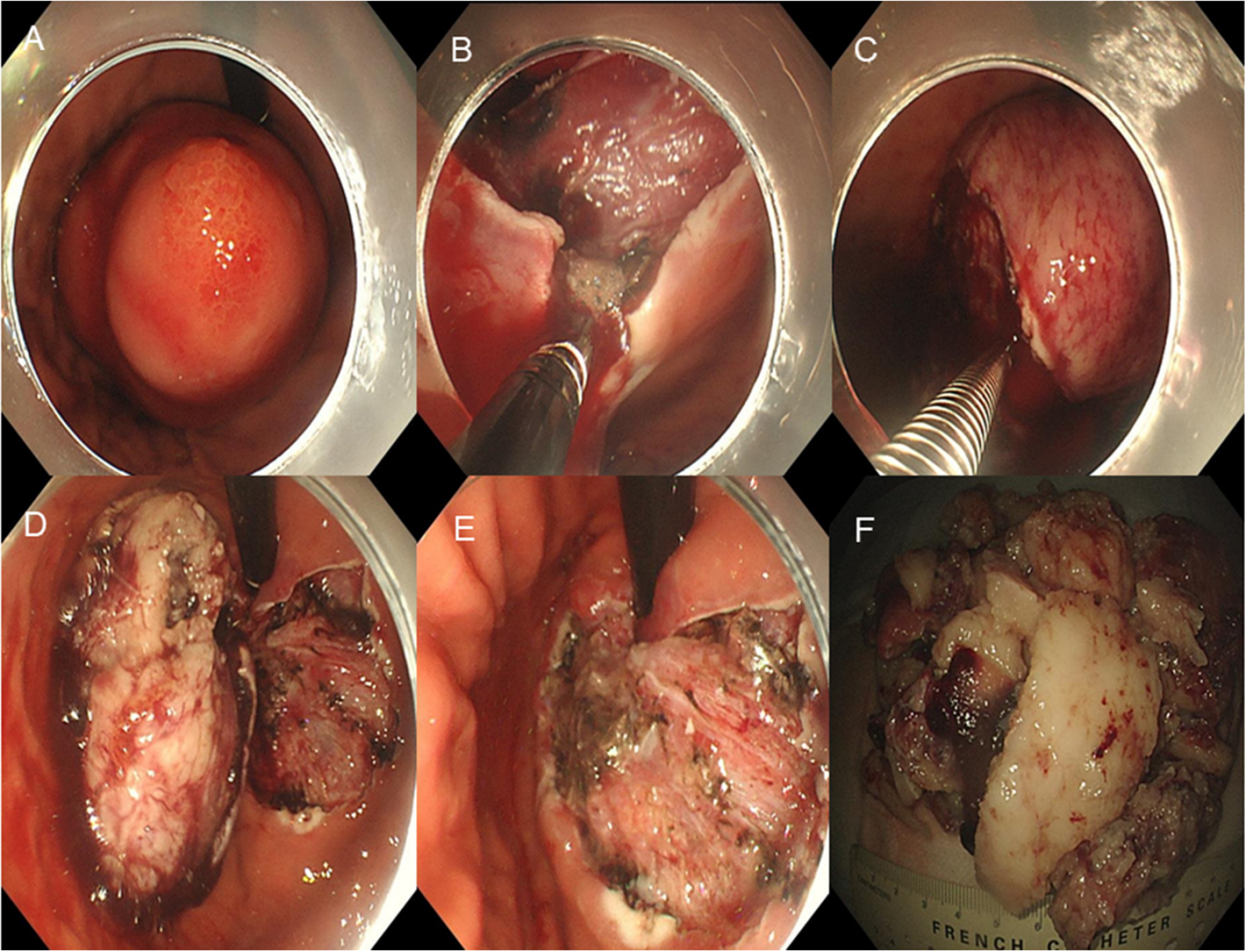
**a** Endoscopic features of the submucosal tumor; **b**, **c** and **d** During endoscopic submucosal excavation (ESE); **e** Endoscopic features after ESE; **f** Gross features of the specimen from ESE

ESE procedure was performed as follows: (1) Argon knife was used to mark the border of the gastric stromal tumor; (2) Saline containing methylene blue and epinephrine was injected into multiple parts of the submucosa outside the marker; (3) A needle knife was used to cut through the mucous membrane; (4) Submucosa was circumferentially cut using the needle knife and an insulated tip knife was used to cut the mucosa along the lateral edge of the marker. Finally, the tumor was resected en bloc manner without bleeding, and an intact pseudocapsule was retained; (5) The wound was treated with thermal biopsy forceps to prevent delayed bleeding; (6) Stone fragments and traps were used to cut the tumor into small pieces, and finally foreign body mesh bags were used to remove the tumor from the body (Fig. 2). The operating time was 90 min.

**Fig. 3:**
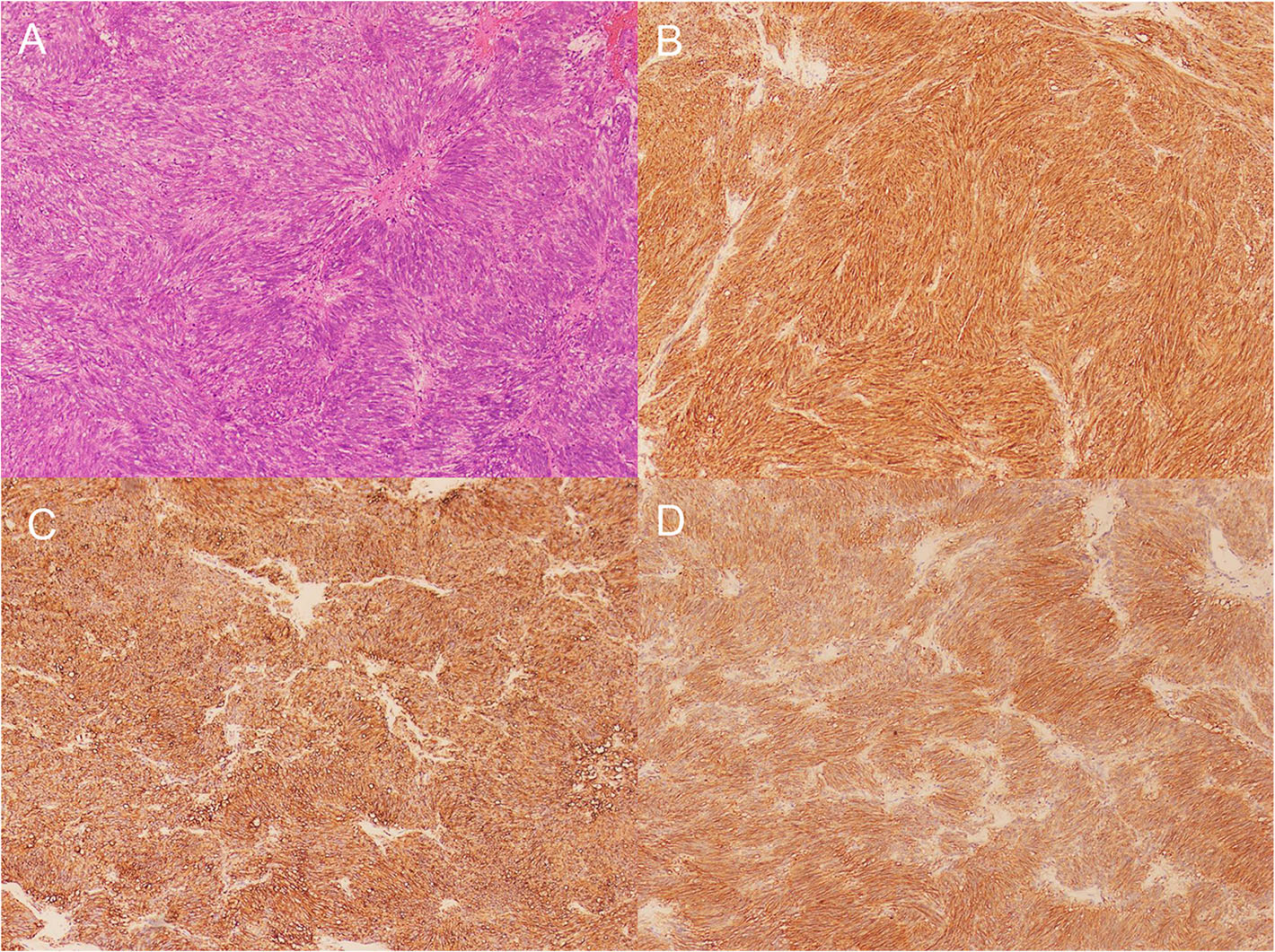
Microscopic examination revealed a spindle-cell neoplasm (**a** hematoxylin and eosin, × 100), and the tumor cells were positive for CD117 (**b** × 100) and CD34 (**c** × 100) and dog-1 (**d** × 100)

**Fig. 4:**
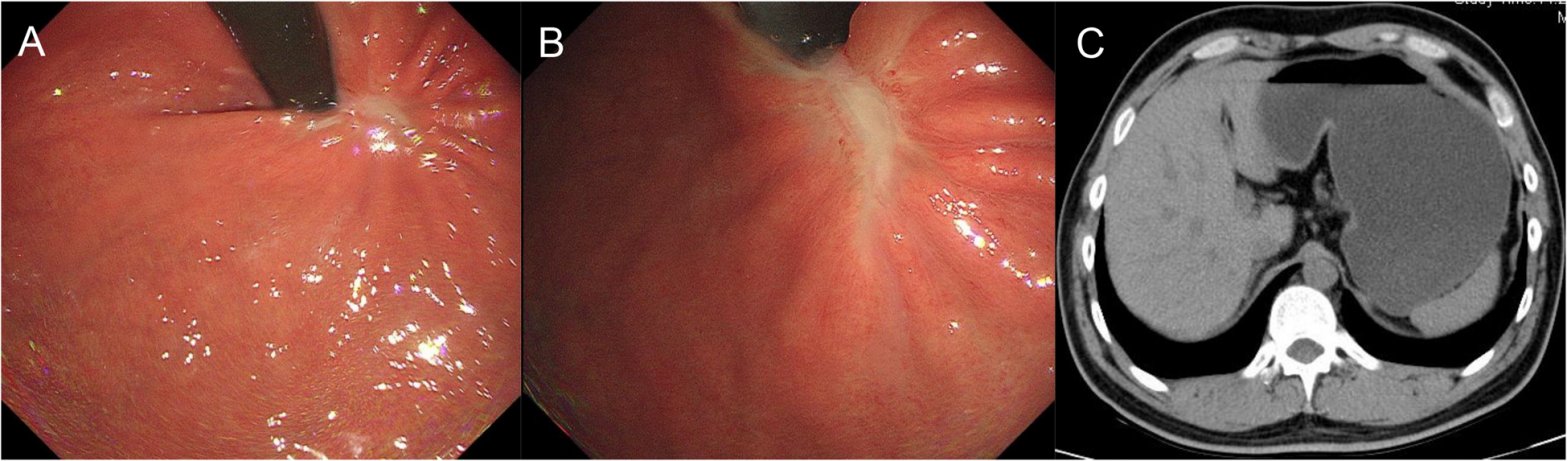
Upper gastrointestinal endoscopy (**a** and **b**) and abdominal CT examination (**c**) were performed 4 months after endoscopic treatment

Histopathological examination confirmed that the tumor was a GIST of spindle cell subtype, and the mitotic index was less than 5 mitotic figures per 50 high power fields. Immunohistochemical analysis revealed that the tumor cells were positive for CD117, Dog-1 and CD34 and negative for SMA, S-100, and Desmin, and the positive rate of ki-67 was 3% (Fig. 3). The specimens were GISTs larger than 5 cm, with less than 5 mitosis per 50 high-power fields, which classified the patient into an intermediate-risk group according to the National Institutes of Health (NIH) consensus criteria for risk stratification of GISTs [3]. Detection of gene mutations included exon 9, 11, 13, 14, 17, and 18 of c-kit gene and exon 12 and 18 of PDGFRA gene. The results showed that exon 11 of c-kit gene was mutated (c.1679 T>G, p. V560G) (42.3%). We recommended that patients should take 1 year of imatinib as adjuvant therapy [4]. Endoscopy showed the healed incision 4 months later. Upper gastrointestinal endoscopy and abdominal CT examination were performed 4 months after endoscopic treatment (Fig. 4), and no signs of recurrence were found. During the follow-up of 8 months, the patient had a normal diet and no serious complications.

## Discussion and conclusions

GISTs are common in the stomach, followed by the intestine. The clinical manifestation of GISTs is related to tumor size, location, and growth pattern, and generally, the symptoms are insidious. Surgical resection is the preferred treatment for localized and potentially resectable GISTs.

Open surgery is a traditional treatment of GISTs. The indications for laparoscopic surgery have been expanding in recent years [5]. However, laparoscopic resection is not indicated for tumors located at the EGJ or near the pyloric ring. For GISTs near the EGJ, due to the limitation of operating range of stapler, the resected specimen is often larger. In our case, the GIST was located on the side of the lesser curvature of the stomach. LWR operation is very difficult, and severe gastric structural changes and stenosis often occur after LWR or open surgery, leading to gastric dysfunction and gastroesophageal reflux. We performed ESE to prevent severe stenosis and reduce the deformity of the EGJ. We removed the stromal tumor completely, and the gastric wall was not perforated, thus avoiding the spread of the tumor cells to the abdominal cavity.

Preoperative administration of Gleevec may reduce the size of the tumor, enabling the surgeons to completely remove the tumor during surgery. Our patient, however, refused to take Gleevec preoperatively for personal reasons and he wanted to remove the tumor as soon as possible. Because the tumor was huge, we could not completely remove it out of the body after the resection. Therefore, we used stone fragments and traps to cut the tumor into small pieces and finally removed the tumor out of the body with a foreign body mesh bag. This procedure was approved by our institution, and informed consent was given by the patient. However, this might have made the judgement of margin involvement impossible. The pseudocapsule was kept intact. Pathological examination and gene detection were also performed. Pathological analysis indicated that the degree of malignancy was intermediate. Patients with GIST with the c-kit exon 11 mutation have reported the best response to imatinib therapy [6].

In summary, the outcome of our patient with a GIST treated by ESE is satisfactory, which avoided excessive resection of healthy gastric wall. ESE is a safe, simple, and effective method for resection of GISTs, especially large stromal tumors located at the EGJ. This also raises a question whether it is feasible to remove the tumor in pieces in stomach, which remains to be proved by more studies.

## Data Availability

Data sharing is not applicable to this article as no datasets were generated or analyzed during the current study.

## Abbreviations

CT: Computed tomography
EGJ: Esophagogastric junction
ESE: Endoscopic submucosal excavation
GIST: Gastrointestinal stromal tumor
GISTs: Gastrointestinal stromal tumors
LWR: Laparoscopic wedge resection
NIH: National Institutes of Health
